# Reconstructing CNV genotypes using segregation analysis: combining pedigree information with CNV assay

**DOI:** 10.1186/1297-9686-42-34

**Published:** 2010-08-12

**Authors:** John M Henshall, Vicki A Whan, Belinda J Norris

**Affiliations:** 1 CSIRO Livestock Industries, FD McMaster Laboratory Chiswick, Armidale, 2350, NSW, Australia; 2 CSIRO Livestock Industries, Queensland Bioscience Precinct, St Lucia 4067, Queensland, Australia

## Abstract

**Background:**

Repeated blocks of genome sequence have been shown to be associated with genetic diversity and disease risk in humans, and with phenotypic diversity in model organisms and domestic animals. Reliable tests are desirable to determine whether individuals are carriers of copy number variants associated with disease risk in humans and livestock, or associated with economically important traits in livestock. In some cases, copy number variants affect the phenotype through a dosage effect but in other cases, allele combinations have non-additive effects. In the latter cases, it has been difficult to develop tests because assays typically return an estimate of the sum of the copy number counts on the maternally and paternally inherited chromosome segments, and this sum does not uniquely determine the allele configuration. In this study, we show that there is an old solution to this new problem: segregation analysis, which has been used for many years to infer alleles in pedigreed populations.

**Methods:**

Segregation analysis was used to estimate copy number alleles from assay data on simulated half-sib sheep populations. Copy number variation at the Agouti locus, known to be responsible for the recessive self-colour black phenotype, was used as a model for the simulation and an appropriate penetrance function was derived. The precision with which carriers and non-carriers of the undesirable single copy allele could be identified, was used to evaluate the method for various family sizes, assay strategies and assay accuracies.

**Results:**

Using relationship data and segregation analysis, the probabilities of carrying the copy number alleles responsible for black or white fleece were estimated with much greater precision than by analyzing assay results for animals individually. The proportion of lambs correctly identified as non-carriers of the undesirable allele increased from 7% when the lambs were analysed alone to 80% when the lambs were analysed in half-sib families.

**Conclusions:**

When a quantitative assay is used to estimate copy number alleles, segregation analysis of related individuals can greatly improve the precision of the estimates. Existing software for segregation analysis would require little if any change to accommodate the penetrance function for copy number assay data.

## Background

With the increasing resolution at which genomes can be examined has come the recognition that variation in genome structure is common and affects more nucleotides per genome than the sequence variation found in single nucleotide polymorphisms (SNP) [1-3]. Copy number variation (CNV) in DNA, defined as insertions, deletions and duplications larger than 1 kb, is an important component of this structural variation. Recent publications document the contribution of CNV to genetic diversity in humans [2,4-6] and human disease [7-9]. CNV has been shown to contribute to phenotype in model organisms [9-11] and to important production and disease traits in domesticated livestock species [12-15]. Current technologies to assay a CNV genotype (genome copy number) and its corresponding alleles have limitations [8,16]. Distinguishing among genomes that have multiple DNA copies (> 4-5 copies) is imprecise while SNP that might 'tag' copy number alleles through linkage disequilibrium are usually only found for relatively simple diallelic CNV [2,3,6,17,18]. Typically, assays attempt to quantify the total number of copies in diploid DNA and cannot discriminate between, for example, an individual homozygous for a two-copy allele and a heterozygous individual carrying one- and three-copy alleles. In many cases, this is not an important limitation as copy number alleles have additive dosage effects, but in other cases it is important, for example when only one copy number allele is associated with disease. To resolve individual alleles, data on related individuals can be analyzed concurrently. Pedigree information applies Mendelian constraints to the allowable sets of copy number alleles in related individuals. These constraints have been exploited using Bayesian graphical models [19] to infer copy number alleles, and hidden Markov model based methods [20,21] to find *de novo *CNV and infer copy number alleles.

The Mendelian constraints underlying analyses such as those noted above have been well studied in the area of segregation analysis. Originally the term "segregation analysis" referred to the determination of the mode of inheritance of a phenotype but in recent decades it has come to include the inference of genotypic probabilities in pedigreed populations. The peeling algorithm of Elston and Stewart [22] is applicable in both cases. In small pedigrees, and in those without inbreeding loops, this algorithm produces unbiased estimates of allelic probabilities. A number of approaches have been described that are computationally feasible for larger pedigrees with inbreeding loops, including iterative peeling [23,24], cutting loops [25] and Markov Chain Monte Carlo (MCMC) methods [26,27]. MCMC methods remain an active area of research (e.g. [28]).

The data required for single locus segregation analysis are four-fold: 1) a pedigree relating individuals to each other; 2) phenotypes on some individuals in the population; 3) a penetrance function expressing the probabilities of phenotype based on genotype; and 4) estimates of the frequencies of the genotype alleles in the population. Probabilities of mutation can also be incorporated. Assays that return estimates of the number of copies in diploid DNA can be thought of as a phenotypic measurement, and if assumptions are made about the distribution of the variation around the expected value of the assay then it is relatively straightforward to derive the appropriate penetrance function. Software to perform computations for segregation analysis is available and may include options to estimate penetrance functions as well as to estimate allelic probabilities (e.g. [29]).

In this paper, we demonstrate that segregation analysis is an old solution to a new problem, by applying a segregation analysis method to simulated data to explore the inference of copy number alleles at the Agouti locus in sheep. The recessive self-colour black condition in domestic sheep has been studied for many years, with allele *A*^*wt*^, responsible for white fleece, known to be dominant to allele *A*^*a *^[30,31], responsible for the dark fibre colour. It has been confirmed that the association is due to variation in the agouti region [32] and recently Norris and Whan [13] have shown that a tandem gene duplication/deletion is responsible. Allele *A*^*wt *^is a number of different alleles, having two or more copies of a 190 kb DNA segment including the agouti signalling protein (ASIP) coding region, while allele *A*^*a *^has a single copy of the region with a non-functional ASIP promoter. Furthermore, Norris and Whan [13] have described an asymmetric competitive PCR copy number assay for the number of copies in diploid DNA. We will use *A*^*c1*^, *A*^*c2 *^... to refer to alleles with one copy, two copies and so on, with allele *A*^*a *^being equivalent to our allele *A*^*c1 *^and allele *A*^*wt *^being replaced by our alleles (*A*^*c2*^, *A*^*c3*^, *A*^*c4 *^...).

In this study, we demonstrate that segregation analysis methods are well suited to the inference of copy number alleles, and that if knowledge on the actual allele configurations is sufficiently important then the utility of a quantitative assay can be greatly improved through the incorporation of relationship data. On small datasets, our methods can be implemented in readily available software such as Mendel [29], accounting for the uncertainty in the individual assay results through the penetrance function. With minor modifications, existing software for large datasets could also be used. While we restrict our analyses to half-sib families, our approach is general and could be applied to any data set with both pedigree and quantitative copy number assay components.

## Methods

### Assay parameters and allele frequency estimation

The characteristics of the assay are reported in Norris and Whan [13]. In an asymmetric competitive PCR assay, DNA amplified from the junctions between copies is compared to the DNA amplified from the junctions and from the 5' breakpoint region. For diploid DNA with a copy number count *n*, the expected value of the assay is the ratio $\frac{n-2}{n}$, as there are *n *- 2 junctions and two copies of the 5' breakpoint. As diploid DNA has at least two copies (at least one on each chromosome) this ratio takes values from the series (0/2, 1/3, 2/4, 3/5 ...) which asymptotically approaches unity. It is important to note that since the assay is quantitative, variation occurs around the expected ratios. The magnitude of the variation can differ among laboratories, and even among batches of samples in one laboratory, so we have treated it as unknown and conducted analyses for a range of values. As a lower limit, we chose a CV of 3% (which equates to a standard deviation of 0.01 for the class with an expected ratio of 1/3). This figure is a little smaller than that derived empirically from ranking 111 assay samples and estimating the mean and standard deviation for the 35 samples that were extremely unlikely to be from a class other than that with an expected ratio of 1/3, the most easily distinguished class. The CV of 3% was used as the lower limit since it is achievable with real data, but we also conducted analyses assuming CV values of 6% and 9%. The expected ratios and standard deviations considered are summarized in Table 1.

**Table 1:** Expected ratios and standard deviations of the assay

		Standard Deviation of Assay		
		
Copies	Expected Ratio	CV = 3%	CV = 6%	CV = 9%
2	0.000	NA	NA	NA
3	0.333	0.010	0.020	0.030
4	0.500	0.015	0.030	0.045
5	0.600	0.018	0.036	0.054
6	0.667	0.020	0.040	0.060
7	0.714	0.021	0.043	0.064
8	0.750	0.023	0.045	0.068
9	0.778	0.023	0.047	0.070
10	0.800	0.024	0.048	0.072
11	0.818	0.025	0.049	0.074
12	0.833	0.025	0.050	0.075

The assay is not required for sheep with two copies as they are phenotypically black and easily identified

From the expected ratios and standard deviations, and assuming a distribution for the variation around the expected value, the probability of each copy number count can be estimated for each observed assay value. We assumed a normal distribution and estimated copy number count probabilities for a population of 87 phenotypically white Merino ewes coming from commercial sale yards. These are summarized in Table 2A. To estimate the allele frequencies that produced this distribution of copy number counts we minimized the X^2 ^statistic obtained by comparing the vector of copy number count frequencies with the frequencies expected given a vector of frequencies for alleles segregating in Hardy-Weinberg equilibrium. Black Merino sheep in wool flocks are commonly culled soon after birth and thus are never presented in a commercial sale yard, so to avoid ascertainment bias we excluded the *A*^*c1*^/*A*^*c1 *^class when calculating the X^2 ^statistic. Table 2B contains the estimated allele frequencies.

**Table 2:** Estimated copy number frequencies (A) and allele frequencies (B) derived from assay results

A. Estimated Copy Number Frequencies		B. Estimated Allele Frequencies	
**Copies**	**Frequency**	**Allele**	**Frequency**

2	0.00	*A*^*c1*^	0.10
3	0.08	*A*^*c2*^	0.43
4	0.29	*A*^*c3*^	0.41
5	0.34	*A*^*c4*^	0.04
6	0.23	*A*^*c5*^	0.00
7	0.04	*A*^*c6*^	0.02
8	0.02	*A*^*c7*^	0.00

Data are from 87 white Merino ewes, and a CV of 3.0% for the assay scores was assumed

### Penetrance function

For black sheep, the genotype is known to be *A*^*c1*^/*A*^*c1 *^with certainty and conversely, for sheep with genotype *A*^*c1*^/*A*^*c1 *^the phenotype is known to be black wool with certainty. The assay is not relevant for these animals. For white sheep, the penetrance function relates to the assay rather than to the phenotype. That is, the penetrance function is the probability of returning a particular assay value given the genotype. Again under the assumption of normality, this is proportional to the height of the normal distribution relevant to the underlying genotype. Table 3 contains an example of the mapping of an assay ratio of 0.675 onto a grid representing the penetrance function for an individual, using standard deviations for a CV of 3%.

**Table 3:** Copy number probabilities and penetrance values for an assay value of 0.675

Copies	3	4	5	6	7	8	9
Probability	0.000	0.000	0.000	0.838	0.159	0.003	0.000
**Penetrance Values**							

		**Copy number allele from dam**					
		
		***A***^***c1***^	***A***^***c2***^	***A***^***c3***^	***A***^***c4***^	***A***^***c5***^	***A***^***c6***^
		
Copy number allele from sire	*A*^*c1*^	0.000	0.000	0.000	0.000	0.838	0.159
	*A*^*c2*^	0.000	0.000	0.000	0.838	0.159	0.003
	*A*^*c3*^	0.000	0.000	0.838	0.159	0.003	0.000
	*A*^*c4*^	0.000	0.838	0.159	0.003	0.000	0.000
	*A*^*c5*^	0.838	0.159	0.003	0.000	0.000	0.000
	*A*^*c6*^	0.159	0.003	0.000	0.000	0.000	0.000

The CV of the assay was assumed to be 3%; the penetrance values are proportional to the probability of an assay value of 0.675 given the genotype

### Simulation

As the stud Merino sheep industry is likely to use a test for the genotype at the Agouti locus, small, highly selected and relatively closed flocks were simulated. In each replicate, 10 studs were simulated, each mating five rams to 200 ewes each year. Selection was on a trait uncorrelated to Agouti, except for the last generation when no homozygous *A*^*c1*^/*A*^*c1 *^animal was selected. In the absence of evidence to the contrary, we assumed that the genotype at the Agouti locus had no effect on fitness other than through artificial selection. The simulation was run for 20 years. In the founder populations, allele frequencies at the Agouti locus were as in Table 2B. There was limited exchange of genetics between studs, with one outside ram and four home-bred rams used by each stud each year. The intention behind this relatively complicated structure was to simulate populations in which cohorts of ewes were related, with variations in the frequencies of the various Agouti alleles between studs. Parents and progeny of the last generation were assumed to be available for assay. Using the known simulated copy number count, an assay value was simulated using the means and standard deviations described above. The whole simulation was repeated 20 times, producing 1,000 half-sib families (20 replicates × 10 studs × 5 rams) for analysis. The distributions of allele frequencies in the final generations of the simulated populations are displayed in Table 4.

**Table 4:** Frequencies of alleles in the final generation of the simulated populations

Frequency Allele	0.0:0.1	0.1:0.2	0.2:0.3	0.3:0.4	0.4:0.5	0.5:0.6	0.6:0.7	0.7:0.8	0.8:0.9
A^c1^	0.79	0.05	0.08	0.08	0.01	0.00	0.00	0.00	0.00
A^c2^	0.07	0.14	0.14	0.11	0.18	0.16	0.07	0.08	0.04
A^c3^	0.04	0.13	0.14	0.10	0.19	0.16	0.07	0.11	0.06
A^c4^	0.89	0.04	0.03	0.03	0.00	0.00	0.00	0.00	0.00
A^c5^	1.00	0.00	0.00	0.00	0.00	0.00	0.00	0.00	0.00
A^c6^	0.96	0.01	0.02	0.01	0.00	0.00	0.00	0.00	0.00

### Relationships between assayed individuals

In our simulation study, we performed the assay on half-sib groups of animals and their parents, ignoring relationships between parents. This is equivalent to "cutting" inbreeding loops in a relatively naive but systematic way, rather than the more sophisticated approaches such as in [25]. We chose this simple approach for a number of reasons, but primarily because we performed many analyses on replicated datasets and therefore needed a fast execution time. However, we believe that the design is justified. In commercial and stud sheep flocks, large half-sib families are usual and although deeper pedigree data is usually available (especially on the male side), it may not be without errors. In half-sib families, most of the power in the method comes from the confidence with which the sire's genotype can be estimated, which then adds confidence to estimates for progeny.

### Software and analysis

Genotype probabilities were estimated using a restricted implementation of the Elston-Stewart algorithm [22], restricted in that it operated only on half-sib families. This restriction allowed a very fast execution time enabling the investigation of a wide range of scenarios. The software was validated by comparing the estimated probabilities for test half-sib families to probabilities estimated using the software package Mendel [29]. The allele frequencies assumed for the parents are those shown in Table 2B, that is, in the analysis we used the frequencies that were used in the simulation. Each dataset was evaluated six times, with one of the three formulas for the standard deviation of the assay (CV = 3%, 6%, 9%) used to simulate the assay values, and either the correct CV or one half of the correct CV used for the analysis. From the final generation of the simulated populations, families of 1, 2, 4, 8, 16 or 32 half-sib progeny were chosen. Assay results were made available for either progeny only, progeny and sire, or progeny, sire and dams. Genotype probabilities were estimated and compared to the simulated copy number alleles.

## Results

Table 5 contains the results for a lamb analyzed without pedigree data. This is not quite the same as relying on assay results alone to estimate the genotype, as population allele frequencies are still used in the analysis. Without these there is no power at all to declare an animal non-carrier. In all cases, when a lamb was declared to be a carrier with greater than 95% certainty it had the genotype *A*^*c1*^/*A*^*c2 *^and in all cases when a lamb had the genotype *A*^*c1*^/*A*^*c2 *^it was declared to be a carrier with over 99.9% certainty.

**Table 5:** Frequencies of estimated probabilities of being a carrier

Status	P < 0.001	P < 0.01	P < 0.05	P > 0.95	P > 0.99	P > 0.999
carrier	0.00	0.00	0.07	0.48	0.48	0.48
non-carrier	0.07	0.25	0.71	0.00	0.00	0.00

For a lamb assayed and analysed without including pedigree data, given actual status (carrier or non-carrier); the assay CV simulated and CV used in the analysis were both 3%

The effect of jointly analyzing half-sib families of varying size is displayed in Table 6. The analyses correspond to a situation where the sire and his half-sib progeny are assayed, and the coefficient of variation (CV) of the assay output is 3%. The most important application of a test in an industrial genetic improvement program is the identification of sheep that are non-carriers. We found that with a family size of one, only 46% of non-carriers were identified as non-carriers with a 99% probability. However, this value is almost twice that achieved without genotyping the sire, as in Table 5. Increasing the family size improved the power, and with family sizes of 16, 75% of non-carriers can be declared clear with a 99% probability. Increasing the family size beyond 16 has only a small effect on power, unless the goal is to achieve 99.9% probabilities of being clear.

**Table 6:** Frequencies of estimated probabilities of being a carrier

Status	Half-sibs	P < 0.001	P < 0.01	P < 0.05	P > 0.95	P > 0.99	P > 0.999
carrier	1	0.00	0.01	0.04	0.48	0.48	0.48
carrier	2	0.00	0.01	0.04	0.48	0.48	0.48
carrier	4	0.00	0.01	0.04	0.50	0.50	0.50
carrier	8	0.00	0.00	0.02	0.58	0.54	0.53
carrier	16	0.00	0.00	0.01	0.66	0.62	0.57
carrier	32	0.00	0.00	0.01	0.72	0.70	0.66
non-carrier	1	0.20	0.46	0.69	0.00	0.00	0.00
non-carrier	2	0.28	0.47	0.69	0.00	0.00	0.00
non-carrier	4	0.35	0.57	0.73	0.00	0.00	0.00
non-carrier	8	0.46	0.68	0.79	0.00	0.00	0.00
non-carrier	16	0.63	0.75	0.81	0.00	0.00	0.00
non-carrier	32	0.72	0.78	0.82	0.00	0.00	0.00

For progeny, given actual status (carrier or non-carrier) and number of half-sibs in family; the assay CV simulated and CV used in the analysis were both 3% and the progeny and sire in each family were assayed

False positives (declaring non-carriers to be carriers) are absent and false negatives (declaring carriers to be non-carriers) are less frequent as family size increases. This is an important result since in industrial applications, this second form of error has a much greater adverse impact. The fact that we do not find proportions of type II errors in accordance with appropriate p-value threshold (for example, 5% of type II errors in the case of a P > 0.95 threshold) is not unexpected, just as we do not expect to identify 95% of carriers with a 95% probability.

Table 7 displays results obtained when exploring the effect of assaying progeny only, or progeny and the sire, or progeny, sire and dam. A family size of 16 half-sibs and a CV of 3% for the assay were used. Even if the sire is not assayed, in 62% of replicates a carrier sire is identified with greater than 99% probability. This improves when the sire is assayed, and if the dams are also assayed almost all the carrier sires are detected. The results are more or less symmetrical, in that the probabilities of declaring a non-carrier sire to be a non-carrier are similar to the probabilities of declaring a carrier sire to be a carrier. For the progeny, the power to detect carriers and non-carriers is lower than for sires and even lower for dams, and if the dam is not assayed there is no power at all to declare her a non-carrier.

**Table 7:** Frequencies of estimated probabilities of being a carrier

Status	Pedigree	Assay	P < 0.001	P < 0.01	P < 0.05	P > 0.95	P > 0.99	P > 0.999
carrier	sire	p	0.00	0.01	0.03	0.71	0.62	0.58
carrier	sire	ps	0.00	0.00	0.01	0.88	0.85	0.80
carrier	sire	psd	0.00	0.00	0.00	0.98	0.98	0.96
carrier	progeny	p	0.00	0.01	0.03	0.56	0.52	0.50
carrier	progeny	ps	0.00	0.00	0.01	0.66	0.62	0.57
carrier	progeny	psd	0.00	0.00	0.02	0.73	0.71	0.68
carrier	dam	p	0.00	0.00	0.00	0.15	0.09	0.06
carrier	dam	ps	0.00	0.00	0.00	0.26	0.22	0.15
carrier	dam	psd	0.00	0.00	0.03	0.78	0.74	0.69
non-carrier	sire	p	0.37	0.67	0.84	0.00	0.00	0.00
non-carrier	sire	ps	0.84	0.93	0.96	0.00	0.00	0.00
non-carrier	sire	psd	0.98	0.99	0.99	0.00	0.00	0.00
non-carrier	progeny	p	0.40	0.61	0.77	0.00	0.00	0.00
non-carrier	progeny	ps	0.63	0.75	0.81	0.00	0.00	0.00
non-carrier	progeny	psd	0.80	0.83	0.90	0.00	0.00	0.00
non-carrier	dam	p	0.00	0.00	0.00	0.00	0.00	0.00
non-carrier	dam	ps	0.00	0.00	0.00	0.00	0.00	0.00
non-carrier	dam	psd	0.58	0.69	0.85	0.00	0.00	0.00

Given actual status (carrier or non-carrier), position in pedigree (sire, dam or progeny), and assay strategy (p = progeny only, ps = progeny and sires, psd = progeny, sires and dams); the assay CV simulated and CV used in the analysis were both 3% and 16 half-sibs in each family were assayed

In Table 8, the effect of the precision of the assay is examined, for family sizes of 16 half-sibs assayed along with their sire. Considering first the situation where the CV used in the analysis is the same as the CV used in the simulation, and again focusing on non-carriers, increasing the CV of the assay decreases the power to declare non-carriers to be clear. This is particularly true if the goal is to achieve a less than 0.1% probability of being a carrier. When an underestimate of the CV is used in the analyses it is not unexpected that the power to declare non-carriers to be clear is improved. This is because reducing the CV reduces the proportion of individuals with ambiguous results, and as most individuals are non-carriers, in most cases overconfidence in the precision of the assay does not result in an error. However, for individuals that are carriers, underestimating the CV results in an increased probability of being declared clear by error.

**Table 8:** Frequencies of estimated probabilities of being a carrier

Status	CVsim	CVan	P < 0.001	P < 0.01	P < 0.05	P > 0.95	P > 0.99	P > 0.999
carrier	0.030	0.015	0.00	0.01	0.02	0.68	0.65	0.61
carrier	0.030	0.030	0.00	0.00	0.01	0.66	0.62	0.57
carrier	0.060	0.030	0.01	0.02	0.04	0.61	0.58	0.55
carrier	0.060	0.060	0.00	0.01	0.03	0.55	0.51	0.48
carrier	0.090	0.045	0.01	0.02	0.06	0.56	0.53	0.51
carrier	0.090	0.090	0.00	0.01	0.04	0.46	0.38	0.25
non-carrier	0.030	0.015	0.68	0.76	0.80	0.00	0.00	0.00
non-carrier	0.030	0.030	0.63	0.75	0.81	0.00	0.00	0.00
non-carrier	0.060	0.030	0.48	0.64	0.74	0.00	0.00	0.00
non-carrier	0.060	0.060	0.33	0.59	0.76	0.00	0.00	0.00
non-carrier	0.090	0.045	0.32	0.52	0.69	0.01	0.00	0.00
non-carrier	0.090	0.090	0.14	0.44	0.69	0.00	0.00	0.00

For progeny, given actual status (carrier or non-carrier), CV used in the simulation (CVsim) and CV used in the analysis (CVan); the sire and 16 progeny in each family were assayed

## Discussion

At the Agouti locus in sheep, colour phenotype is affected by a recessive, single copy allele, which cannot always be uniquely identified using the available assay. Furthermore, the assay has almost no power to determine that an animal is free of the undesirable single copy allele. Exploiting the family structures common in sheep flocks, across the scenarios examined, the joint analysis of half-sib families resulted in a modest increase in the power to declare individuals as carriers of the undesirable *A*^*c1 *^allele. More importantly in a selective breeding environment, including family data resulted in a large increase in the power to declare individuals as non-carriers of the *A*^*c1 *^allele.

The improvement is dramatic: from 7% of non-carrier lambs being identified as clear at the 99.9% level when a lamb is assayed and analyzed alone (last row, Table 5), to as many as 80% of non-carrier lambs being declared clear at the same threshold when the sire, dams and lambs for a half-sib family of 16 are assayed and analyzed together (4^th ^last row, Table 7). This is achieved at a cost of assaying 33 related individuals, and looks a better strategy than assaying 32 half-sib lambs and their sire but not their dams (last row, Table 6), especially as ewes are generally used over a number of years and would not need to be re-assayed.

Provided that the precision of the assay is not overestimated (i.e. the CV underestimated) in formulating the penetrance function, the joint analysis of half-sib families does not increase the proportion of false negatives. On the contrary, it reduces the proportion, from 7% at a 5% threshold for lambs analyzed alone, to as few as 1% if 16 half-sibs are analyzed together. As noted earlier, the assignment of a non-carrier status to carriers is most undesirable in industrial applications of the assay. A ram, sold as a non-carrier, that subsequently produces progeny exhibiting the self colour black condition, can adversely affect the reputation of the stud selling the ram, and the reputation of the assay. Furthermore, if the ram is used only in flocks that are clear of the *A*^*c1 *^allele, then the undesirable allele can become established in the ewe population. Thus it might take several generations before this is detected when another carrier ram is used.

In this paper, we have focused on an example from livestock, the self colour black condition in sheep which means we have assumed large half-sib families and restricted ourselves to analyzing these in isolation. These analyses are very quick and the analysis of high-throughput quantitative CNV assay data for half-sib families would be feasible using this method. However, a half-sib family structure is not a requirement for a segregation analysis, and even for family sizes of one or two half-sibs with the sire being also assayed, power was increased to a higher value than when analyzing an individual alone. Readily available segregation analysis software makes optimal use of all pedigree links in estimating allele probabilities, and is suitable for copy number allele calling in human pedigrees and other pedigrees with smaller family sizes.

In our simulation study, we assumed that parameters relating to penetrance function were known. Specifically, we assumed allele frequencies and parameters for a normal distribution for the error associated with the quantitative assay. These were estimated from a sample of unrelated sheep, but could be estimated from the population of interest. This aspect of segregation analysis was not applied in this study, but software such as Mendel [29] can be used for this purpose. In our study, we also investigated an already known copy number variant, identified from an experimental population designed to uncover the cause of the recessive black condition in Merino sheep. In most cases an experimental population will not be available. Using segregation analysis to search for *de novo *CNV affecting quantitative traits would be similar to using hidden Markov model based approaches [20,21], but from a different statistical perspective. To apply segregation analysis software for general pedigrees to high-density genomic data would likely be computationally prohibitive but software such as that used here for half-sib families might be feasible.

## Conclusions

The precision of copy number allele estimates from quantitative assay data in pedigreed populations is greatly increased if the pedigree information is used in the estimation, and segregation analysis methods based on the peeling algorithm are well suited to this application. In the case of the Agouti locus and the recessive self-colour black condition, where the purpose of the test is to identify animals to use as parents, the proportion of lambs correctly identified (at the 99.9% level) as non-carriers increased from 7% when the lambs were analysed alone to 80% when the lambs were analysed in families. Any segregation analysis software can be used provided that the appropriate penetrance function is specified.

## Competing interests

The authors declare that they have no competing interests.

## Authors' contributions

JMH wrote the software for simulating and analysing the datasets, carried out the analyses and drafted the manuscript. VAW conducted the molecular genetic studies and contributed to the manuscript. BJN contributed to the design of the study and to the manuscript. All authors have read and approved the final manuscript.

## References

[B1] Feuk L Carson AR Scherer SW Structural variation in the human genome Nat Rev Genet 2006 7 85 97 10.1038/nrg1767 16418744

[B2] Redon R Ishikawa S Fitch KR Feuk L Perry GH Andrews TD Fiegler H Shapero MH Carson AR Chen WW Global variation in copy number in the human genome Nature 2006 444 444 454 10.1038/nature05329 17122850 PMC2669898

[B3] Sebat J Major changes in our DNA lead to major changes in our thinking Nat Genet 2007 39 S3 S5 10.1038/ng2095 17597778

[B4] Conrad DF Andrews TD Carter NP Hurles ME Pritchard JK A high-resolution survey of deletion polymorphism in the human genome Nat Genet 2006 38 75 81 10.1038/ng1697 16327808

[B5] Goidts V Cooper DN Armengol L Schempp W Conroy J Estivill X Nowak N Hameister H Kehrer-Sawatzki H Complex patterns of copy number variation at sites of segmental duplications: an important category of structural variation in the human genome Hum Genet 2006 120 270 284 10.1007/s00439-006-0217-y 16838144

[B6] McCarroll SA Hadnott TN Perry GH Sabeti PC Zody MC Barrett JC Dallaire S Gabriel SB Lee C Daly MJ Altshuler DM Common deletion polymorphisms in the human genome Nat Genet 2006 38 86 92 10.1038/ng1696 16468122

[B7] Lupski JR Genomic rearrangements and sporadic disease Nat Genet 2007 39 S43 47 10.1038/ng2084 17597781

[B8] McCarroll SA Altshuler DM Copy-number variation and association studies of human disease Nat Genet 2007 39 S37 42 10.1038/ng2080 17597780

[B9] Henrichsen CN Chaignat E Reymond A Copy number variants, diseases and gene expression Hum Mol Genet 2009 18 R1 8 10.1093/hmg/ddp011 19297395

[B10] Dopman EB Hartl DL A portrait of copy-number polymorphism in Drosophila melanogaster Proc Natl Acad Sci USA 2007 104 19920 19925 10.1073/pnas.0709888104 18056801 PMC2148398

[B11] Jackson AN McLure CA Dawkins RL Keating PJ Mannose binding lectin (MBL) copy number polymorphism in Zebrafish (D-rerio) and identification of haplotypes resistant to L-anguillarum Immunogenetics 2007 59 861 872 10.1007/s00251-007-0251-5 17943278

[B12] Pielberg G Olsson C Syvanen AC Andersson L Unexpectedly high allelic diversity at the KIT locus causing dominant white color in the domestic pig Genetics 2002 160 305 311 11805065 10.1093/genetics/160.1.305 PMC1461930

[B13] Norris BJ Whan VA A gene duplication affecting expression of the ovine ASIP gene is responsible for white and black sheep Genome Res 2008 18 1282 1293 10.1101/gr.072090.107 18493018 PMC2493430

[B14] Wright D Boije H Meadows JRS Bed'hom B Gourichon D Vieaud A Tixier-Boichard M Rubin CJ Imsland F Hallbook F Andersson L Copy Number Variation in Intron 1 of SOX5 Causes the Pea-comb Phenotype in Chickens Plos Genetics 2009 5 6 e1000512 Epub 2009 Jun 12 10.1371/journal.pgen.1000512 19521496 PMC2685452

[B15] Pielberg GR Golovko A Sundstrom E Curik I Lennartsson J Seltenhammer MH Druml T Binns M Fitzsimmons C Lindgren G Sandberg K Baumung R Vetterlein M Stromberg S Grabherr M Wade C Lindblad-Toh K Ponten F Heldin CH Solkner J Andersson L A cis-acting regulatory mutation causes premature hair graying and susceptibility to melanoma in the horse Nat Genet 2008 40 1004 1009 10.1038/ng.185 18641652

[B16] Seo B-Y Park E-W Ahn S-J Lee S-H Kim J-H Im H-T Lee J-H Cho I-C Kong I-K Jeon J-T An accurate method for quantifying and analyzing copy number variation in porcine KIT by an oligonucleotide ligation assay BMC Genetics 2007 23;8 81 10.1186/1471-2156-8-81 PMC2228321 18036219

[B17] Hinds DA Kloek AP Jen M Chen X Frazer KA Common deletions and SNPs are in linkage disequilibrium in the human genome Nat Genet 2006 38 82 85 10.1038/ng1695 16327809

[B18] Locke DP Sharp AJ McCarroll SA McGrath SD Newman TL Cheng Z Schwartz S Albertson DG Pinkel D Altshuler DM Eichler EE Linkage disequilibrium and heritability of copy-number polymorphisms within duplicated regions of the human genome Am J Hum Genet 2006 79 275 290 10.1086/505653 16826518 PMC1559496

[B19] Kosta K Sabroe I Goke J Nibbs RJ Tsanakas J Whyte MK Teare MD A Bayesian approach to copy-number-polymorphism analysis in nuclear pedigrees Am J Hum Genet 2007 81 808 812 10.1086/520096 17847005 PMC2227930

[B20] Wang K Li MY Hadley D Liu R Glessner J Grant SFA Hakonarson H Bucan M PennCNV: An integrated hidden Markov model designed for high-resolution copy number variation detection in whole-genome SNP genotyping data Genome Res 2007 17 1665 1674 10.1101/gr.6861907 17921354 PMC2045149

[B21] Wang K Chen Z Tadesse MG Glessner J Grant SFA Hakonarson H Bucan M Li MY Modeling genetic inheritance of copy number variations Nucleic Acids Res 2008 36 21 e138 10.1093/nar/gkn641 18832372 PMC2588508

[B22] Elston RC Stewart J A general model for the genetic analysis of pedigree data Hum Hered 1971 21 523 542 10.1159/000152448 5149961

[B23] Van Arendonk JAM Smith C Kennedy BW Method to estimate genotype probabilities at individual loci in farm animals Theor Appl Genet 1989 78 735 740 10.1007/BF00262571 24225836

[B24] Janss LLG Van Arendonk JAM Van der Werf JHJ Computing approximate monogenic model likelihoods in large pedigrees with loops Genet Sel Evol 1995 27 567 579 10.1186/1297-9686-27-6-567

[B25] Stricker C Fernando RL Elston RC An algorithm to approximate the likelihood for pedigree data with loops by cutting Theor Appl Genet 1995 91 1054 1063 10.1007/BF00223919 24169996

[B26] Lange K Sobel E A Random Walk Method for Computing Genetic Location Scores Am J Hum Genet 1991 49 1320 1334 1746559 PMC1686461

[B27] Guo SW Thompson EA Monte Carlo Estimation of Mixed Models for Large Complex Pedigrees Biometrics 1994 50 417 432 10.2307/2533385 8068842

[B28] Abraham KJ Totir LR Fernando RL Improved techniques for sampling complex pedigrees with the Gibbs sampler Genet Sel Evol 2007 39 27 38 10.1186/1297-9686-39-1-27 17212946 PMC2739431

[B29] Lange K Cantor R Horvath S Perola M Sabatti C Sinsheimer J Sobel E Mendel version 4.0: a complete package for the exact genetic analysis of discrete traits in pedigree and population data sets Am J Hum Genet 2001 69 504 504 10.1086/322739 11462172 PMC1235481

[B30] Hayman RH Cooper DW The frequency of pigmented sheep in the Australian Merino Wool Technol Sheep Breed 1965 12 81 85

[B31] Brooker MG Dolling CHS Pigmentation of sheep. I. Inheritance of pigmented wool in the Merino Aust J Agric Res 1965 16 219 228 10.1071/AR9650219

[B32] Parsons YM Fleet MR Cooper DW The Agouti gene: a positional candidate for recessive self-colour pigmentation in Australian Merino sheep Aust J Agric Res 1999 50 1099 1103 10.1071/AR98099

